# Complete Genome Sequences of *Betanodavirus* from Australian Barramundi (*Lates calcarifer*)

**DOI:** 10.1128/MRA.00081-19

**Published:** 2019-04-18

**Authors:** Kelly Condon, Shaun Bochow, Ellen Ariel, Terrence L. Miller

**Affiliations:** a College of Public Health, Medical and Veterinary Sciences, James Cook University, Townsville, Queensland, Australia; b Centre for Sustainable Tropical Fisheries and Aquaculture, James Cook University, Townsville, Queensland, Australia; c Department of Primary Industries and Regional Development Western Australia, DPIRD Diagnostics and Laboratory Services, Perth, Australia; Queens College

## Abstract

The complete RNA-1 and RNA-2 genome sequences of *Betanodavirus* were obtained from Australian barramundi (*Lates calcarifer*). Phylogenetic analyses revealed that the sequences have closest homology to the red spotted grouper nervous necrosis virus (RGNNV) species and share between 91 and 98% homology with the other two published complete/near-complete sequences of isolates from Australian fish.

## ANNOUNCEMENT

Members of the genus *Betanodavirus* cause the disease viral nervous necrosis (VNN), which is synonymous with vacuolating/viral encephalopathy and retinopathy (VER) (1). The disease has been reported from wild and cultured freshwater and marine fish from all continents except South America and Antarctica. The National Centre for Biotechnology Information (NCBI) database (www.ncbi.nlm.nih.gov) contains over 1,200 nucleotide accessions of betanodavirus sourced from over 220 fish species and over 30 countries. VNN has been recognized in Australia since 1988, when it first caused significant mortalities in hatchery-reared larval barramundi, *Lates calcarifer* (2). Of the 15 betanodavirus sequences from Australian fish species present in the NCBI database, there are only three complete or near-complete genome sequences (3, 4).

Diseased *L. calcarifer* juveniles were collected from an aquaculture hatchery in Queensland, Australia. The fish displayed mass mortality and clinical signs typical of VNN, including loss of appetite, darkened color, erratic and spiral swimming, and hyperinflation of the swim bladder. Betanodavirus was detected using reverse transcriptase quantitative PCR (RT-qPCR) analysis on eye and brain tissues (3). Further PCR, cloning, and sequence analysis were conducted. Eyes and brains were removed from clinically affected fish, homogenized, and subjected to RNA extraction (Roche High Pure viral nucleic acid extraction), and cDNA was synthesized using random hexamers (Bioline Tetro cDNA synthesis kit). PCR analysis using primers that targeted RNA-1 and RNA-2 were completed (3). PCR amplicons were purified and cloned using the pCR4-TOPO TA vector system. Plasmid DNA was extracted and submitted to Macrogen, Inc., and the Australian Genome Research Facility for analysis by Sanger sequencing. Sequencing data and alignments were analyzed using the default parameters in Geneious v.9.8.1 and the BLAST (5) in NCBI. Overlapping fragments from RNA-1 were aligned to form a contig (3,098 nucleotides [nt]) using Geneious and applying the red spotted grouper nervous necrosis virus (RGNNV) type species as the reference genome. The complete sequence of RNA-2 (1,036 nt) was obtained from direct cloning (3).

The RNA-1 sequence contained the mRNA encoding RNA-dependent RNA polymerase and B1 and B2 protein motifs characteristic of *Betanodavirus* RNA-1 genomes. The RNA-2 sequence contained the complete open reading frame (ORF) encoding the viral coat protein. Alignment of the sequence against type sequences of the four currently recognized *Betanodavirus* taxa indicated that the sequences have highest homology to the RGNNV type species.

Comparative analysis at the nucleotide level indicated that the RNA-1 sequence was 95 to 99% homologous to the sequences within the RGNNV genotype and 96 to 98% homologous to the only two complete RNA-1 sequences obtained from Australian strains (NCBI accession numbers GQ402010 and GQ402012).

Comparative analysis at the nucleotide level of RNA-2 indicated 99% homology to the sequence obtained from the original RGNNV isolate from China in 2000 (NCBI accession number AY744705) and 91 to 98% homology to the only 3 complete/near-complete RNA-2 sequences from Australian strains (NCBI accession numbers GQ402013, GQ402011, and KT390714). This report adds to the genome record of betanodavirus strains endemic to Australia and continues to demonstrate the remarkable trend of conservation of the RGNNV genome sequence across time, host species, and geographic location.

### Data availability.

The gene sequences have been deposited in GenBank under the accession numbers MH181161 (RNA-1) and MH017207 (RNA-2).

## References

[1] World Organisation for Animal Health. 2018 Chapter 2.3.12, Viral encephalopathy and retinopathy *In* Manual of diagnostic tests for aquatic animals. World Organisation for Animal Health, Paris, France http://www.oie.int/index.php?id=2439&L=0&htmfile=chapitre_viral_encephalopathy_retinopathy.htm.

[2] Glazebrook JS, Heasman MP, de Beer SW 1990 Picorna-like viral particles associated with mass mortalities in larval barramundi, *Lates calcarifer* Bloch. J Fish Dis 13:245–249. doi:10.1111/j.1365-2761.1990.tb00780.x.

[3] Hick P, Whittington RJ 2010 Optimisation and validation of a real-time reverse transcriptase-polymerase chain reaction assay for detection of betanodavirus. J Virol Methods 163:368–377. doi:10.1016/j.jviromet.2009.10.027. 19891987

[4] Agnihotri K, Pease B, Chong R 2016 Molecular analysis of RNA1 and RNA2 sequences from a betanodavirus isolated from giant grouper (*Epinephelus lanceolatus*) in Australia. Virol Rep 6:25–31. doi:10.1016/j.virep.2016.05.001.

[5] Altschul SF, Gish W, Miller W, Myers EW, Lipman DJ 1990 Basic local alignment search tool. J Mol Biol 215:403–410. doi:10.1016/S0022-2836(05)80360-2. 2231712

